# Extraction Methods, Quantitative and Qualitative Phytochemical Screening of Medicinal Plants for Antimicrobial Textiles: A Review

**DOI:** 10.3390/plants11152011

**Published:** 2022-08-02

**Authors:** Elvino Nortjie, Moses Basitere, Doice Moyo, Pardon Nyamukamba

**Affiliations:** 1 Bioresource Engineering Research Group, Department of Chemical Engineering, Cape Peninsula University of Technology, Bellville, Cape Town 7535, South Africa; elvinonortjie@gmail.com; 2 Academic Support Programme for Engineering in Cape Town (ASPECT), Centre for Higher Education Development, University of Cape Town, Rondebosch, Cape Town 7700, South Africa; 3 Technology Station Clothing and Textiles, Symphony Way, Bellville, Cape Town 7535, South Africa; moyod@cput.ac.za (D.M.); nyamukambap@cput.ac.za (P.N.)

**Keywords:** antimicrobial agents, textile finishings, extractions, solvents, phytochemical screening, qualitative analysis, quantitative analysis

## Abstract

Medicinal plants are the product of natural drug discoveries and have gained traction due to their pharmacological activities. Pathogens are everywhere, and they thrive in ideal conditions depending on the nutrients, moisture, temperature, and pH that increase the growth of harmful pathogens on surfaces and textiles. Thus, antimicrobial agents and finishes may be the solution to the destruction of pathogens. This review article presents an analysis of various aspects of producing antimicrobial finishings, the microorganisms, their mechanism of attachment to natural and synthetic fibre, the effect of microbial growth, and the principle and mechanism of the microbial activity of the medicinal plants. Furthermore, the extraction methods, qualitative and quantitative phytochemical evaluations of antimicrobial efficacy, and developments of antimicrobial treated textiles using various agents are covered in this review.

## 1. Introduction

Antibiotics play a vital role in fighting bacterial infections, but antibacterial resistance has caused havoc in the healthcare and pharmaceutical sector that accelerates socio-economic losses [1]. Multidrug Resistance is said to increase by 10 million deaths per year by 2050 [1,2]. Biological screening, separation of the phytochemicals, and clinical trials of the medicinal plants have advanced over the years unfolding the secrets of ancient herbal remedies [3]. Traditional medicine is effective in dealing with diseases caused by bacteria or oxidative stress [4,5,6]. 

Natural compounds have been extensively explored for new drug discoveries [7] Humanity has always been fascinated by natural compounds from pre-biotic, microbial, plant, and animal sources. Extracts of different parts of plants contain bioactive compounds that fight against diseases such as alkaloids, steroids, tannins, glycosides, volatile oils, fixed oils, resins, phenols, terpenoids, and flavonoids [8] The phenolic phytochemicals from plants play a key role as antimicrobial agents [6,9] Antimicrobial agents decay the protein components of the cell wall, disrupting the work of enzymes and DNA and RNA replication [10] Table 1 shows a selection of plants, their phytochemicals responsible for antimicrobial activity, and their applications.

## 2. Textiles and Microorganisms

Textiles are carriers of microorganisms and are subjected to the growth of microorganisms, such as bacteria and fungi, depending on the food, acidic pH, temperature, time, oxygen, and moisture [11]. Bacteria interrelates with fibres in phases, from initial attachment onto fibres to the growth and damage to the fibres [12]. Cotton is one of the ideal natural fibre fabrics for the growth of pathogens than polyester. Neely [21]. has shown the survival of several gram-positive bacteria (*Staphylococcus aureus*, *Enterococcus faecalis*) on standard hospital fabrics made of 100% cotton clothing, 100% cotton terry towels, 60%/40% cotton/polyester-scrub suits and lab coats, and 100% polyester drape.

A study by Neely [21] showed the growth of bacteria within 48 h, most bacterial growth survived at least a day, and some survived more than 90 days. Natural fibre textiles are more prone to microbial growth and could lead to the spread of infections [14]. A study by Gupta [22] reports that the attachment of the bacteria onto the fabric is dependent on the characteristics of the fabric, the contact time of the microbe onto the fabric, surface roughness, and moisture retention for natural and synthetic fibres reacting differently to microbial growth [2]. Natural fibres are more prone to microbial attack because they retain water easily. Microbial growth on synthetic fibres like polyester is slower due to their polymer backbone [12].

## 3. Requirements, Modes of Antimicrobial Action of Antimicrobial Agents

The ideal antimicrobial treatment for textiles must be effective against a broad spectrum of pathogens but exhibit low toxicity to the user. It must be cost-effective, durable to launder, and not alter the quality or appearance of the textile [23].

A study by Gao et al., [24] reported that microbes are microscopic organisms that exist as unicellular, multicellular, or cell clusters. They consist of an outermost cell wall that constitutes polysaccharides. The cell wall maintains the integrity of cellular components and shields the cell from the extracellular environment. Beneath the cell wall is a semi-permeable membrane that encloses intracellular organelles and multiple enzymes and nucleic acids. The enzymes are responsible for the chemical reactions within the cell, followed by the storage of nucleic acid genetic information of the organism. The purpose of antimicrobial agents is to destroy the cell wall or alter cell membrane permeability, denature proteins, inhibit enzyme activity, or inhibit lipid synthesis so that the cell does not survive.

The modes of antimicrobial action of antimicrobial agents define the existence of antimicrobial agents. The antimicrobial agents target mainly the cell wall, and cell membrane, denature protein, inhibit enzyme activity, and inhibit lipid synthesis. There are various classes of antimicrobial agents that possess different mechanisms of action against microbes. Table 2 shows the different antimicrobial classes, the mechanisms of action, and the activity spectrum, respectively [25]. Adapted with permission from Ref. [25]. 2014, Dr Patricia Tille.

Textiles are regarded as the only barrier between humans and pathogens. Plant-based extracts and materials provide an efficient and natural microbial resistivity. Antimicrobial textiles are essential in the apparel, commercial, and healthcare sector [26]. A study by Vastrad et al. [27] reported on the evaluation of total phenolic content and flavonoid content using leaf extracts (eucalyptus and lemongrass) with methanol, ethanol, chloroform, and distilled water extract indicated the potential of antimicrobial application of textiles. The antimicrobial agents and finishing on textiles may allow the re-use of face masks, and clothing, reducing PPE kits in health care, reducing domestic laundering that may lead to a reduction in water consumption, curtailing the worldwide pandemic, global warming, and environmental degradation.

## 4. Pre-Treatment and Processing of Biomass

### 4.1. Drying of Biomass

The selection of pre-treatment and processing methods may influence the reduction in extraction time, an increase in extraction yield, quality of the biological compounds, and reduction in input energy [28]. The drying of any biomass inhibits microbial growth [18], and it aids in the longer shelf life and transportation costs due to the weight and space of dry products [29,30,31]. Drying can affect the phytochemical components of the thermally sensitive components [29,32,33,34], and the process can also contribute to improved conservation of the bioactive compounds against oxidative [35] and enzymatic activities [36] and spoilage bacteria [30,37,38], enabling cellular destruction [28,30,35]. There are many different drying methods, e.g., thermal through natural convection (shade and open sun drying), forced convection (oven drying, solar drying, and heat pump drying), freeze-drying, greenhouse drying, microwave drying, and infrared drying [28].

The freeze-drying method retains the bioactive compounds of the dried product due to minimal thermal damage to the cell tissue, thermolabile compounds, and its porous surface, enabling increased penetration of solvents [30,35,39] Olive leave extracts pre-treated with a hot air drier at 120 °C showed higher phenolic recovery compared to freeze-drying (loss of polyphenols reached up to 39% in dry weight). Freeze-drying shows great potential in the extraction of the total phenolic content [35,40].

Ahmad-Qasem [35] reported that temperature plays a key role in the drying process as it may be beneficial or unfavourable to the microstructure of the biomass and the use of hot air drying at a high temperature. The study by Ahmad-Qasem [35] also reported better extraction efficacy of some phenolic compounds in olive leaves when compared to samples dried at lower temperatures and by drying at a moderate to low temperature may need a longer drying time to reach the desired moisture content of the biomass.

### 4.2. Choice of Solvents

The solvent selection is crucial in determining the bioactive compounds of plants used for extractions. Ideal extraction solvent properties include low toxicity, evaporating easily at low temperatures, having good solubility of the target compound, and being sufficiently volatile. The factors affecting the selection of solvents are the rate of extraction, diversity of compounds extracted, ease of handling of extracts, and the cost-effectiveness of the extraction solvents and targeted compounds. Plants consist of various bioactive compounds with varying polarities. Various techniques have been developed and used to obtain pure compounds determining the structure and biological activity [41]. Many solvent extractions have been done to obtain phytochemical compounds for their activity against pathogens. Different phytochemicals have different structural features and consist of different phytochemical compounds as well as action mechanisms as described below:

- Phenols and polyphenols are obtained from acetone and ethanol solvent extractions which consist of C_3_ sidechain, hydroxyl groups and a phenol ring e.g., catechol, epicatechin, cinnamic acid that has antimicrobial, anthelmintic, and antidiarrheal activity. The mechanism of action of polyphenols binds to proteins (adhesins), inhibits enzyme-substrate deprivation, complexes with the cell wall, makes intestinal mucosa more resistant and reduces secretion, increases the supply of digestible proteins by animals by forming protein complexes in the rumen, and causes a decrease in gastrointestinal-tract metabolism [42,43].
- Chloroform, methanol, and ethanol solvents extract mainly quinones. They consist of aromatic rings, two ketone substitutions e.g., hypericin that has antimicrobial activity. The mechanisms of action of quinones inactivate enzymes, complex with the cell wall, and bind to proteins (adhesins) [42,43].
- Ethanol and water mainly extract tannins which consist of polymeric phenols e.g., ellagitannin which has antimicrobial anthelmintic and antidiarrheal activities. The mechanism of action of tannins allows the binding of proteins (adhesins), inhibits enzyme-substrate deprivation, complexes with the cell wall, makes intestinal mucosa more resistant and reduces secretion, increases the supply of digestible proteins by animals by forming protein complexes in the rumen, and causes a decrease in gastrointestinal-tract metabolism [42,43].
- Chloroform solvents extract mainly flavonoids which consist of phenolic structure, a carbonyl group, hydroxylated phenols C_3_ –C_5_ unit linked to an aromatic ring, flavones and a +3-hydroxyl group that has antimicrobial, anthelmintic and antidiarrheal activity. The mechanism of action of flavonoids is complex with the cell wall, binds to proteins (adhesins), inhibits the secretion of autocoids and prostaglandins and inhibits contractions caused by spasms [42,43].
- Ether solvent extracts mainly coumarins and it consists of phenols made up of fused benzenes e.g., warfarin with antimicrobial activity. The mechanism of action of coumarins allows the interaction with eukaryotic DNA [42,43].
- Water, ethanol, chloroform, and ether solvents extract mainly terpenoids which consist of fatty acids and acetate units with antimicrobial activity. The mechanism of action of terpenoids inhibits the release of autocoids and prostaglandins [42,43].
- Lectins and polypeptides can be extracted by water which consists of mainly extracts proteins e.g., mannose-specific agglutinin, and fabatin that has antimicrobial activity. The mechanism of action of lectins and polypeptides blocks viral fusion or adsorption. [42,43].
- Alkaloids can be extracted by ethanol and ether solvents which consist of heterocyclic nitrogen compounds e.g., berberine, piperine, palmatine and tetrahydropalmatine which has antimicrobial, anthelmintic and antidiarrheal activity. The mechanism of action of alkaloids inhibits the secretion of autocoids and prostaglandins and possesses anti-oxidating effects, thus reducing nitrate generation, which is useful for protein synthesis and suppresses the transfer of sucrose from the stomach to the small intestine. [42,43].
- Glycosides are mainly obtained when extracted by ethanol solvent, which consists of sugar plus a non-carbohydrate moiety e.g., amygdalin which has antidiarrheal activity. The mechanisms of action of glycosides inhibit the secretion of autocoids and prostaglandins [42,43].

Saponins can be extracted by methanol, water, and hydro-alcoholic 70 % methanol which consists of amphipathic glycosides e.g., vina-ginsenosides R5-R6 with antidiarrheal activity. The mechanism of action of saponins inhibits histamine release in-vitro [42,43]. The selection, identification, and collection of plants are critical for phytochemical studies. It is crucial to have the plants identified by a plant specialist. Many plants are selected through either traditional means by humans or by investigations based on reports of their biological properties. During extraction, solvents diffuse in the plant material and dissolve compounds with similar polarity. The plant’s bioactive chemicals depend on the plant material origin, conditions of the plant it has grown or cultivated in, moisture content, and particle size of the plant parts. The different extraction methods will also affect the composition of the secondary metabolites of the extracts namely, type of extraction, time of extraction, temperature and nature of the solvent, solvent concentration, and polarity.

The determination of biologically active compounds from plant materials is crucial and dependent on the type of solvent used [44]. Solvents are selected based on their availability, low toxicity, boiling point, ease of evaporation, and solvent polarity [45,46]. The FAO/WHO Expert Committee reported seventeen solvents that are allowed and regarded as safe to use for food and personal-care products.

## 5. Microorganisms

Resistance to antibiotics has become a serious problem globally. ESKAPE are multidrug-resistant pathogens such as *Enterococcus faecium*, *Staphylococcus aureus*, *Klebsiella pneumoniae*, *Acinetobacter baumannii*, *Pseudomonas aeruginosa*, and *Enterobacter* species who are responsible for Hospital-Acquired Infections (HAI). New antibiotics have been produced over the years. The resistance by the ESKAPE pathogens to the drugs has accelerated tremendously [47]. A Priority Pathogen List (PPL) was released by the WHO in 2016 as a guide to research, discovery, and development of new antibiotics globally [48]. Pathogens occupy the surfaces of fabrics depending on the contact time, moisture retention, and surface roughness. Staphylococcus aureus and Escherichia coli pathogens cause hospital infections leading to pneumonia and sepsis. It is, therefore, important to keep track of the availability of alternative medicinal plants and herbs to conquer this challenge [49]. The discovery of new drugs that can be mastered with the use of plant extracts is a hoard of a spectrum of secondary metabolites [50,51,52,53,54,55,56].

### 5.1. Enterococcus faecium

*Enterococcus faecium* is a Gram-positive bacterium that causes infections; it is increasingly resistant compared to *Vancomycin-resistant Enterococci faecium* [57,58]. *E. faecium* lives in the gut microbiome of animals [59,60]. Food is an excellent hideout for the strains to remain dormant [61]. Treatment is dependent on second-line antibiotics [62]. Urinary tract infections, bacteraemia, and endocarditis are caused by this bacterium [60].

### 5.2. Staphylococcus aureus

*Staphylococcus aureus* is a Gram-positive bacterium prevalent on the human skin, particularly in immune-compromised individuals. This bacterium causes infections on medical implants and forms biofilms that make it extremely difficult to treat with antibiotics. The Methicillin-resistant Staphylococcus aureus developed resistance against β-lactam antibiotics [58]. Community Associated-Methicillin-Resistant Staphylococcus aureus lineages are associated with skin and soft tissue infections. The Methicillin-resistant Staphylococcus Aureus strains are associated with pneumonia and bloodstream infections [63].

### 5.3. Klebsiella pneumonaie

*Klebsiella pneumonaie* is a Gram-negative bacterium that causes urinary tract infections septicaemia, surgical wound infections, pneumonia, endocarditis, pyogenic liver abscess cystitis, and endogenous endophthalmitis [64]. The Cephalosporin- and carbapenem-class antibiotics have been the base treatment for *Enterobacterales* infections, such as *Klebsiella pneumoniae.* The efficacy of the antibiotics is compromised by the widespread acquisition of genes and encoding enzymes that aid in the respective resistance to these critical drugs [65].

### 5.4. Acinetobacter baumannii

*Acinetobacter baumannii* is a Gram-negative bacterium that is more common in hospital settings [66]. It is aerobic and non-fermenting pleomorphic. This bacterium can resist dehydration. It forms biofilms, surface adhesins, and secretions systems that help this bacterium thrive in its environment [67]. The infection rates of *A. baumannii* are low compared to the ESKAPE pathogens [68].

### 5.5. Pseudomonas aeruginosa

*Pseudomonas aeruginosa* is a Gram-negative bacterium associated with respiratory infections and displays resistance to multiple classes of antibiotics [68]. *P. aeruginosa* grows and colonizes in moist environments, especially in healthcare settings in the context of chronic wounds, respiratory support, or urinary tract devices, immune evasion, and antimicrobial resistance [69].

### 5.6. Enterobacter *spp.*

It’s a Gram-negative bacterium, anaerobic in nature. *Enterobacter aerogenes*, known as *Klebsiella aerogenes* are responsible for the increasing hospital-acquired infections [69]. Immunocompromised individuals are more susceptible to urinary and respiratory tract infections to this bacterium [70].

### 5.7. Escherichia coli

*Escherichia coli* which is not part of the ESKAPE pathogens is the major cause of bloodstream and urinary tract infection (UTI) in both community and health care settings globally. Sepsis is one of the most common manifestations of E. coli urinary tract infection. *E. coli* is the most common Gram-negative bacterial species isolated from blood and urine cultures [71].

### 5.8. Brief Description of the Biocide Agents on the Market

Many biocide agents already exist on the market. They are classified into the following compounds [72]:

Quaternary Ammonium Compounds: These compounds represent a group of compounds. They consist of a subgroup of alkyl linear ammonium compounds, composed of hydrophobic alkyl chain and hydrophilic-counterpart. Quaternary Ammonium Compounds damage cell membranes, modify proteins and inhibits DNA production. They are applied in cotton, polyester, nylon and wool fibres. These compounds are active against a wide range of pathogens but lack physical bonding in textiles.

Triclosan: These compounds are odourless chlorinated bisphenol and improve the durability of laundering. They are active against a wide range of pathogens. They block lipid biosynthesis and hinder the integrity of the cell membranes. They are applied to polyester, nylon, cellulose acetate and polypropylene.

Metals and metallic salts: At low concentrations, they are exceptionally active against pathogens. They generate reactive oxygen species, damaging cellular proteins lipids and DNA. Silver, copper, zinc, and cobalt are used has been widely used as antimicrobial agents and applied to cotton, wool, nylon, and polyester.

Chitosan: is a natural hydrophilic copolymer. It’s a linear polysaccharide that is biocompatible, non-toxic, non-carcinogenic, and antimicrobial. They are applied to cotton, wool, polyester and nylon fibres. The low molecular weight results in inhibiting the synthesis of mRNA, preventing protein synthesis, and the high molecular weight causes leakage of intracellular substances or blocks the transport of essential solutes.

Poly (Hexamethylene Biguanide): These agents are polycationic amines biguanide repeat units separated by aliphatic chains. They interact with membrane phospholipids, resulting in disturbance and the fatal leakage of cytoplasmic materials. They are applied to cotton, nylon, and polyester fibres.

N-halamines: They are heterocyclic organic compounds. N-halamines prevent the cell enzymatic and metabolic processes, causing the consequent microorganism destruction. They are applied in cotton, nylon, polyester and wool fibres and are active against a wide range of pathogens.

Many plant-based compounds with a wide range of antimicrobial activity spectrum have been identified and are commercially available. Table 3 shows the wide range of commercially available antimicrobial agents on the market.

## 6. Extraction Methods for Studying Phytochemicals

### 6.1. Introduction

There are various extraction methods, e.g., solvent extraction, distillation method, pressing, and sublimation. Solvent extraction is the most widely used method where the natural products undergo a process where the solvent penetrates through the plant cell wall and the solute dissolves in the solvents the solute followed by collecting the extract. It has been reported that the size of the plant material, properties of the solvent solid to solvent ratio extraction temperature, and extraction time will affect the extraction efficiency [73,74]. The selectivity of the solvents, solubility, cost and safety play a crucial role in solvent extraction. Solvents with the same polarity as the polarity of the solute will result in a greater yield. High temperature affects dispersion and solubility. High temperatures may result in solvents being lost and extracts with impurities and the degradation of thermolabile compounds. The extraction efficiency increases with extraction time. Increasing time will not affect the extraction. The greater the solvent to solid ratio, the greater the extraction yield [75].

Various extraction methods are used to extract the desired bioactive compounds from the plant materials, e.g., solvent extraction, distillation method, pressing, and sublimation. Solvent extraction is the most widely used extraction method when extracting from plant material.

#### 6.1.1. Cold Extraction

In this extraction process, the plant parts are dried in a controlled environment at low temperatures and milled into a powder and weighed. The powder is added to a beaker with solvents and kept at room temperature for thirty minutes. The contents are shaken every twenty-four hours for seven days. The extract is filtered using Whatman filter paper under vacuum and drying at room temperature in a watch glass dish. The weight of the powder is recorded before and after drying [76].

#### 6.1.2. Plant Tissue Homogenization

Fresh plant parts are grounded in a blender. The solvent is added and shaken vigorously for 5–10 min or left for 24 h followed by filtration of the extract. The filtrate can be dried under reduced pressure and redissolved in the solvent to determine the concentration, or it can be centrifuged for clarification for further studies [44].

#### 6.1.3. Serial Exhaustive Extraction

In this extraction method, the solvent of increasing polarity from a non-polar solvent (hexane) to a polar solvent (methanol) is used to ensure a broad polarity range of compounds being extracted and to prepare crude extracts [44].

#### 6.1.4. Soxhlet Extraction

In this extraction method, solid material is placed in a thimble in the extractor. The solvent is heated until reflux. The vapour rises, and the solvent is condensed and fills up the thimble. The extraction is repeated [77,78].

#### 6.1.5. Maceration

A whole or coarsely powdered plant is soaked in the solvent in a container for a period under continuous mixing until agitation until the biomass matter is dissolved [44].

#### 6.1.6. Decoction

In this extraction method, the plant parts are brought to a boil in water followed by cooling, straining, and passing sufficient cold water through the drug to produce the required volume [77].

#### 6.1.7. Infusion

In this extraction method, the plant parts are macerated with either cold or boiling water [77].

#### 6.1.8. Digestion

In this extraction method, the plant parts are macerated under gentle heating [77].

#### 6.1.9. Percolation

In this extraction method, the raw material is placed in an appropriate amount of solvent for approximately 4 h in a closed container. Additional solvent is added to the top of the raw material and macerated in a closed container for 24 h. The percolator is opened, and the extract is poured out drip-wise. Additional solvent is added until the percolate measures about three-quarters of the required volume of the finished product. The marc is pressed, and the pressed liquid is added to the percolate. Additional solvent is added to produce the required volume, and the mixed liquid is clarified by filtration or by decanting [77].

#### 6.1.10. Sonication

This method uses ultrasound technology to assist in the extraction of the bioactive compounds under frequencies ranging from 20 kHz to 2000 kHz. The ultrasound increases the permeability of cell walls and produces cavitation and ruptures the plant cell wall [77].

#### 6.1.11. Enzymatic Extraction

In this extraction method, enzymes are used to increase the yields during the extraction. Enzymes are used to soften the tissues of biomass and facilitate the degradation of the cells [79].

#### 6.1.12. Microwave-Assisted Extraction

This extraction method uses microwave radiation and solvents to extract bioactive compounds. Microwave energy is generated through microwave radiation that heats the solvents whilst increasing the kinetics of the extraction. Moisture occurs in the plant cells when heat is applied and evaporates. The microwave effect generates pressure on the cell wall and results in cell rupture. Exudation occurs and leads to an increase in extraction yield [79].

#### 6.1.13. Ultrasonic-Assisted Extraction

This is an extraction method using ultrasonic sound waves that pass through the solvent, producing energy by enhancing the diffusion of the solvent into the sample array. The Ultrasonic-Assisted Extraction is cost-effective in terms of the quantity of solvent used, temperature, and time [79].

#### 6.1.14. The Supercritical Fluid Extraction

In this extraction method, supercritical fluids at high temperatures and pressures above the critical values are applied to the extraction material. The pressure is adjusted, and the supercritical fluids return to their gas phase and evaporate without leaving solvent residues [79].

#### 6.1.15. Pressurised Liquid Extraction

This extraction method is conducted under high pressures and temperatures that aid in the high solubility of the compounds in the solvent and result in high diffusion of the solvent into the sample array [79]. Table 4 shows the various extraction methods used when extracting biomass.

### 6.2. Chromatography Techniques

#### 6.2.1. Introduction

Chromatography is a technique used to separate molecules based on their size, shape, and charge. The analyte in the solvent passes through a molecular sieve which leads to its separation. Paper and thin layer chromatography readily provide qualitative information and through which it becomes possible to obtain quantitative data.

#### 6.2.2. Paper Chromatography (PC)

In this technique, a sheet of paper is used to carry out separations which acts as both support as well a medium for separation. The sample is placed near the bottom of the filter paper and the filter paper is placed in the chromatographic chamber with solvent. The solvent moves forward by capillary action carrying soluble molecules along with it. Low porosity paper will produce a slow rate of movement of the solvent and thick papers have increased sample capacity [80]. 

#### 6.2.3. Thin Layer Chromatography (TLC)

This technique is used to separate the samples based on the interaction between a thin layer of adsorbent attached to the plate with low molecular weight compounds. Different adsorbents are used to separate various compounds [80].

#### 6.2.4. Gas Chromatography (GC)

This technique is used to separate volatile compounds. The rate of kinetics for the chemical species is determined through its distribution in the gas phase. Gas chromatography involves a sample being vaporized and injected onto the head of the chromatographic column. The sample is transported through the column by the flow of the inert, gaseous mobile phase. The column itself contains a liquid stationary phase which is adsorbed onto the surface of an inert solid [80].

#### 6.2.5. High-Performance Liquid Chromatography (HPLC)

This technique separates compounds based on their interactions with solid particles of a tightly packed column and the solvent of the mobile phase. The Diode Array Detector measures the absorption spectra of the analytes to aid in their identification of the compounds [80].

### 6.3. Qualitative and Quantitative Phytochemical Screening

#### 6.3.1. Introduction

The study of bioactive compounds encompasses phytochemical and pharmacological approaches [81] Many plant parts contain bioactive components, e.g., bark, leaves, stems, fruits, and seeds [82]. Phytochemicals are chemicals produced by the various parts of the plants namely, alkaloids, flavonoids, terpenoids, steroids, tannins, glycosides, etc. The bioactive compounds have various antimicrobial and antibacterial properties [83]. Qualitative phytochemical screening plays a crucial role in identifying various biochemical compounds produced by plants. The quantification of those metabolites may assist in the extraction, purification, and identification of the bioactive compounds for human use [83]. The preliminary qualitative phytochemical screening is carried out as per standard methods described by Trease & Evans 1989.

##### Detection of Alkaloids

The extracts are dissolved in dilute hydrochloric acid and filtered individually and tested for the presence of alkaloids.

**Mayers test:** The extraction added to the Mayers reagent. A yellow cream precipitate formation indicates the presence of alkaloids.

**Wagner’s test:** Wagner’s reagent is added to the extraction if a brown-reddish brown formation is observed, and it indicates the presence of alkaloids.

Detection of Flavonoids

**Lead acetate test:** A few drops of lead acetate solution is added to the extracts. A yellow-colour precipitate indicates the presence of flavonoids.

**Sulfuric acid test:** A few drops of sulfuric acid are added to the extracts, and the formation of orange colour indicates the presence of flavonoids.

##### Detection of Steroids

A few drops of acetic anhydride are added to the extracts and the formation of violet to blue to green in some samples indicates the presence of steroids.

##### Detection of Terpenoids

**Salkowski’s Test**: Extract of 5 mg of the selected plant part is mixed with 2 mL chloroform and 3 mL concentrated sulfuric acid added carefully to form a layer. A reddish-brown colour indicates the presence of terpenoids.

##### Detection of Anthraquinones

**Bontrager’s Test:** About 5 mg of the extract is boiled with 10% HCl for a few minutes in a water bath. It’s filtered and allowed to cool. An equal volume of CHCl_3_ is added to the filtrate. A few drops of 10% NH_3_ are added to the mixture and heated. The formation of pink colour indicates the presence of anthraquinones.

##### Detection of Phenols

**Ferric chloride test:** A few drops of ferric chloride are added to the 10 mL extract. A bluish-black colour indicates the presence of phenol.

**Lead acetate test:** A few drops of lead acetate solution is mixed with 10 mg extract. A yellow colour indicates the presence of phenol.

##### Detection of Saponins

A 0.5 mg of the extract is mixed vigorously with 5 mL of distilled water. The formation of frothing indicates the presence of saponins.

##### Detection of Tannins

A few millilitres of the extract are mixed with a few millilitres of water and heated in a water bath. The mixture is filtered. Ferric chloride is added to the filtrate. The dark green colour indicates the presence of tannins.

##### Detection of Carbohydrates

A 0.5 mg of the extract is dissolved individually in five ml of distilled water and filtered. The filtrate is used to test the presence of carbohydrates [84].

#### 6.3.2. Quantitative Phytochemical Analysis

##### Estimation of Total Alkaloids

One gram of extract sample is added to a 250 mL beaker, and 200 mL of 10% acetic acid in ethanol is added, covered, and left for settling for 4 h. The extract is filtered and concentrated in a water bath to one-quarter of the original volume. Concentrated ammonium hydroxide is added dropwise to the extract until the precipitation is complete. The solution is allowed to settle, and the precipitate is collected and washed with dilute ammonium hydroxide, followed by filtration. The residue is dried and weighed [85]. 

##### Estimation of Total Flavonoids

A gram of sample is extracted repeatedly with 100 mL of 80% aqueous methanol. The mixture is filtered through Whatman no.1 filter paper into a pre-weighed 250 mL beaker. The filtrate is transferred to a water bath and allowed for evaporation to dryness and followed by weighing off the sample [83].

##### Estimation of Total Phenols

The sample is placed in a beaker and boiled for 15 min with 50 mL of ether for the extraction of phenolic compounds. five mL of the extract is pipetted out into a 50 mL flask followed by the addition of 10 mL of distilled water, 2 mL of ammonium hydroxide solution, and 5 mL of concentrated amyl alcohol. The samples are left to react for 30 min for colour development and read at 505 nm [83].

### 6.4. Textiles Analysis

#### 6.4.1. Biocidal Analysis

The biocidal analysis evaluates the effectiveness of antimicrobial textiles. Several test methods have been established through quantitative antimicrobial tests. The number of microbes present on the finished fabrics can be counted and expressed as a percentage or as a log reduction. The test methods for quantitative determination are ATCC TM100, JIS L1902, AATCC90 percentage reduction, and ISO 20743 shake flask reduction methods [86].

The Parallel Streak Method (AATCC TM147) is a qualitative method used to determine the antibacterial activity of diffusible antimicrobials agents on treated textile materials. The Parallel Streak Method has proven to be effective. This method shows antibacterial activity against both Gram-positive and Gram-negative bacteria. The sterilised agar is dispensed (cooled to 47 °C (117 °F) by pouring 15 mL into each standard (15 × 100 mm) flat bottomed petri dish. Allow agar to gel firmly before inoculating. The inoculum is prepared by transferring 1.0 mL of a 24-h broth culture into 9.0 mL of sterile distilled water containing it in a test tube or small flask. A 4 mm inoculating loop is used, loaded with one loopful of the diluted inoculum and transferred to the surface of the sterile agar plate by making five streaks approximately 60 mm in length, spaced 10 mm apart by covering the central area of a standard petri-dish without refilling the loop. The specimen is pressed onto the agar surface with a sterile spatula. After 18 to 24 h of incubation at 37 °C, the plates are examined for bacterial growth directly underneath the textiles and around the edges of the textiles. If the antimicrobial substance diffuses into the agar, an inhibition area is formed, and its size indicates the effectiveness of the antimicrobial effect or the rate at which the active agent is released [21,24]. AATCC 100 (Suspension Test) is a quantitative antimicrobial test method used to determine the antibacterial activity of the textiles and fabrics against bacteria. The bacterial counts are recorded, and a percent reduction is measured using initial count and remaining count data [24].

#### 6.4.2. Durability Analysis

Durability by washing method (ASTM E3162-18 or AATCC61-2A) is used to determine the durability of laundering. This test method is an accelerated laundering test method to measure the durability of antibacterial agents applied to textiles under simulated home laundering conditions. Ten grams of the coated fabric for laundering is prepared, followed by adding a 500 mL defined detergent solution. Set the washing machine at a temperature of 50 °C under abrasive action using stainless steel balls to simulate five home launderings for a 45-min laundering cycle at 40 revolutions per minute. After each cycle, remove the fabric and rinse with water thoroughly by hand. Repeat, depending on the total number of washes required.

## 7. Conclusions

Plants are a unique source of bioactive compounds with biological activities and medicinal properties. The choice of solvents plays an important role in the extraction of bioactive chemicals. Antimicrobial agents and textile finishes have gained traction over the years. Synthetic antimicrobial agents show great effectiveness against pathogens but cause harm to the environment and human health. More research on plant-based antimicrobial agents and finishing should be done to extend the longevity of the antimicrobial power and durability to laundering on textiles substrates. The rise of “super germs” has become a global health problem due to antibiotic resistance. More research needs to be done on medicinal plants as a source of alternative medicines using unexplored medicinal plants for their bioactive properties and solvents that are generally regarded as safe. There should be more in-depth studies done on the most economical pre-treatment, drying, and extraction methods for future therapeutics.

## Figures and Tables

**Table 1:** Representation of medicinal plant extracts and their applications.

Plant Name	Phytochemicals	Applications
*Sutherlandia fructecens*	Saponins, pinitols, flavonoids, triterpenoids, Cannavanine, cycloartane glycosides, flavonol glycosides, and aminobutyric acid [11].	Wound treatment, cancer treatment, diabetes, skin diseases, rheumatism, urinary tract infection, fever, gonorrhoea, kidney, and liver problems [11].
*Eucomis autumnalis*	Homoisoflavanones, terpenoids, and diben-α-pyrones [12].	Reducing fever, urinary diseases, stomach, lower backaches, and syphilis. *Eucomisautumnalis* issometimes used to induce labour [12].
*Plumbago auriculata*	Tannins, phenols, alkaloids, saponins, flavonoids, plumbagin, α-amyrin, capensisone, and diomuscinone [13].	Treating headaches, warts, skin infections, wounds, and fractures [13].
*Catharanthus roseus*	Vinblastine, deoxyvinblastin, vincoline, cathanranthamine, rosicine, leurosine, vindoline and vincristine [14].	Treating rheumatism, venereal diseases, skin infections, high blood pressure, and diabetes [15].
*Aspalathus linearis*	Spalathin, orientin, isoquercitrin, and luteolinhyperoside [15].	Treat insomnia, stomach cramps, allergies, and digestive problems as well as improve appetite [16].
*Centella asiatica*	Triterpenoids, centellose, medacassoside, triaponosides, flavonoid quercetin, rutin, kaempferol, patuletin, apigenin, polyacetylenes, phenolic acids, sterols [17].	Treating fever, leprosy, syphilis, tuberculosis, leprosy, asthma, epilepsy, mental disorder, and minor wounds. Consumed as a vegetable and used as a spice [17].
*Sclerocarya birrea*	Glucosides, steroids, glycosides, flavonoids, fatty oils, alkaloids, phenols, resins, calcium, and phosphorus [18].	Treating dysentery, rheumatism, malaria, and diarrhoea [18].
*Hypoxis hemerocallidea*	Rooperol, β-sitosterol [19].	Immune booster, purgative, and laxative tonic. Treat tuberculosis, urinary tract infection, infertility, cancer, diabetes, and wounds [19].
*Galenia africana*	Trihydroxyflavanone, trihydroxychalcone, dihydroxychalcone, trihydroxy-3-methoxychalcone [20].	Treat venereal sores, eye infections, asthma, tuberculosis, cough, wounds, skin infections and relieve toothache [20].

**Table 2:** Representation of mechanisms of action of antimicrobial agents [25]. Adapted with permission from Ref. [25]. 2014, Dr Patricia Tille.

Antimicrobial Class	Mechanism of Action	Activity Spectrum
β-lactams	They inhibit cell wall synthesis by binding enzymes in peptidoglycan production	Gram-negative bacteria and gram-positive bacteria could differ with individual antibiotic
Aminoglycosides	Hinders the protein synthesis by binding 30S ribosomal subunits	Gram-negative bacteria and Gram-positive bacteria
Chloramphenicol	Inhibits the protein synthesis by binding 50S ribosomal subunits	Gram-negative bacteria and Gram-positive bacteria
Fluoroquinolones	Inhibits DNA synthesis by binding the DNA gyrase topoisomerase IV	Gram-negative bacteria and gram-positive bacteria, but it could differ with individual antibiotic
Glycylglycines	Inhibits the protein synthesis by binding 50S ribosomal units	A wide spectrum of gram-negative bacteria and gram-positive species
Ketolides	Inhibits protein synthesis by binding 50S ribosomal subunits	Gram-positive cocci including certain macrolide resistance strains and Gram-negative strains
Lipopeptides	Binding and disruption of cell membrane	Gram-positive bacteria including β-lactams and glycopeptides
Nitrofurantoin	The mechanism is unknown and may have bacterial enzyme targets and damaging DNA	Gram-negative bacteria and gram-positive bacteria
Oxazolidinones	Hinders the initiation of protein synthesis by binding 50S ribosomal subunits	Wide variety of Gram-positive bacteria including those resistant antimicrobial classes
Polymyxins	Disrupts cell membrane c	Poor activity against most Gram-positive bacteria. Gram-negative bacteria
Rifampin	Hinders RNA synthesis by binding DNA dependent, RNA polymerase	Gram-positive and certain Gram-negative bacteria
Streptogramins	Hinders the protein synthesis by binding two separate sites on the 50S ribosomal subunit	Gram-positive bacteria
Tetracycline	Inhibits protein synthesis by binding of 30S ribosomal subunit	Gram-negative bacteria and gram-positive bacteria and several intracellular bacterial pathogens
Sulfonamides	Hinders the folic acid pathway, binding the enzyme dihydropteroate synthase	Gram-negative bacteria and gram-positive bacteria
Trimethoprim	Hinders with the folic acid pathway by binding the enzyme dihydrofolate reductase	Gram-negative bacteria and gram-positive bacteria

**Table 3:** Representation of commercially available antimicrobial agents on the market [72].

Product Name	Company	Description
Agion^®^	Sciessent, Beverly, MA, USA	Silver and zeolite-based additive
AlphaSan^®^	Milliken Chemical, Spartanburg, SC, USA	Silver-based additive
BioGaurd^®^	AEGI Microbe Shield, Huntersville, NC, USA	Finishing agent based on 3-trimethoxysilylpropyldimethyloctadecylammonium chloride
Biozac ZS	Zschimmer & Schwarz Mohsdorf GmbH, Burgstadt, Germany	PHMB-based finishing agent
Cosmocil CQ™	Lonza, Basel, Germany	Polyaminopropyl biguanide- based additive
Eosy^®^	Unitika, Osaka, Japan	Finishing agent based on chitosan
Irgaurd^®^ 1000	BASF, Ludwigshafen, Germany	Finishing agent based on triclosan
Irgasan	Sigma Aldrich, St. Louis, MO, USA	Finishing agent based on triclosan
Microban^®^	Microban International, Huntersville, NC, USA	Triclosan-based agent
Reputex™	Lonza, Basel, Germany	PHMB-based finishing agent
Sanigard KC	L. N. Chemical Industries, Maharashtra, India	Finishing agent belonging to the QAC group
Saniguard Nano-ZN	L. N. Chemical Industries, Maharashtra, India	Finishing solution based on aqueous nano-dispersion of zinc oxide
Sanitised^®^	SANITIZED AG, Burgdorf, Germany	Finishing agent based on 3-trimethoxysilylpropyldimethyloctadecylammonium chloride
Silpure^®^	Thomson Research Associates, Toronto, ON, Canada	Silver particles-based finishing agent
Silvadur™	The Dow Chemical Company, Midland, MI, USA	Interpenetrating polymer network with silver ions
SmartSilver^®^	Nanohorizon Inc., Philadelphia, PA, USA	Silver nanoparticles-based agent
Silverion 2400	Pure Bioscience, Inc., El Cajon, CA, USA	Stabilised silver complex-based agent

**Table 4:** Extraction methods used in biomass extractions [75].

Method	Solvent	Temperature	Pressure	Time	Volume Consumed	The Polarity of Natural Products
Maceration	Water, Aqueous and non-aqueous solvents	Room temperature	Atmospheric	Long	Large	Dependent on extracting solvent
Percolation	Water, Aqueous and non-aqueous solvents	Room temperature, occasional heat	Atmospheric	Long	Large	Dependent on extracting solvent
Decoction	Water	Under heat	Atmospheric	Moderate	None	Polar compounds
Reflux extraction	Aqueous and non-aqueous solvents	Under heat	Atmospheric	Moderate	Moderate	Dependent on the extracting solvents
Soxhlet extraction	Organic solvents	Under heat	Atmospheric	Long	Moderate	Dependent on extracting solvent
Pressurised liquid extraction	Water, aqueous and non-aqueous solvents	Under heat	High	Short	Small	Dependent on extracting solvent
Supercritical fluid extraction	CO_2_	Near room temperatures	High	Short	None or small	Non-polar to moderate compounds
Ultrasound-assisted extraction	Water, aqueous and non-aqueous solvents	Room temperature or under heat	Atmospheric	Short	Moderate	Dependent on extracting solvent
Microwave-assisted extraction	Water, aqueous and non-aqueous solvents	Room temperature	Atmospheric	Short	Moderate	Dependent on extracting solvent
Pulsed electric field extraction	Water, aqueous and non-aqueous solvents	Room temperature or under heat	Atmospheric	Short	Moderate	Dependent on extracting solvent
Enzyme assisted extraction	Water, aqueous and non-aqueous solvents	Room temperature or heated after enzyme treatment	Atmospheric	Moderate	Moderate	Dependent on extracting solvent

## Abbreviations

ISO	International Standards Organization
AATCC	American Association of Textile Chemists and Colourists
JIS	Japanese Industrial Standards
PC	Paper Chromatography
TLC	Thin Layer Chromatography
GC	Gas Chromatography
HPLC	High-performance liquid chromatography
QAC	Quaternary Ammonium Compounds
mRNA	messenger Ribonucleic acid
HAI	Health Associated Infections
PPL	Priority Pathogen List
WHO	World Health Organization
RNA	Ribonucleic acid
DNA	Deoxyribonucleic acid
UTI	Urinary Tract Infection
ESKAPE	*Enterococcus faecium*, *Staphylococcus aureus*, *Klebsiella pneumoniae*, *Acinetobacter baumanni*, *Pseudomonas aeruginosa*, *Acinetobacter aerogenes*
